# Role of SDF1/CXCR4 Interaction in Experimental Hemiplegic Models with Neural Cell Transplantation

**DOI:** 10.3390/ijms13032636

**Published:** 2012-02-28

**Authors:** Nagisa Arimitsu, Jun Shimizu, Naruyoshi Fujiwara, Kenji Takai, Erika Takada, Takao Kono, Yuji Ueda, Tomoko Suzuki, Noboru Suzuki

**Affiliations:** 1 Department of Immunology and Medicine, St. Marianna University School of Medicine, Sugao 2-16-1, Miyamae-ku, Kawasaki 216-8511, Japan; E-Mails: arimitsu.na@marianna-u.ac.jp (N.A.); jshimizu@marianna-u.ac.jp (J.S.); drnalf72@marianna-u.ac.jp (N.F.); kj-takai@marianna-u.ac.jp (K.T.); erika-ta@marianna-u.ac.jp (E.T.); yueda@ops.dti.ne.jp (Y.U.); tomoclinic2010@gmail.com (T.S.); 2 Department of Neurosurgery, St. Marianna University School of Medicine, Sugao 2-16-1, Miyamae-ku, Kawasaki 216-8511, Japan; E-Mail: t2kohno@marianna-u.ac.jp

**Keywords:** neural stem/progenitor cells, chemokines, cell migration, chemokine receptor

## Abstract

Much attention has been focused on neural cell transplantation because of its promising clinical applications. We have reported that embryonic stem (ES) cell derived neural stem/progenitor cell transplantation significantly improved motor functions in a hemiplegic mouse model. It is important to understand the molecular mechanisms governing neural regeneration of the damaged motor cortex after the transplantation. Recent investigations disclosed that chemokines participated in the regulation of migration and maturation of neural cell grafts. In this review, we summarize the involvement of inflammatory chemokines including stromal cell derived factor 1 (SDF1) in neural regeneration after ES cell derived neural stem/progenitor cell transplantation in mouse stroke models.

## 1. Introduction

**Figure 1:**
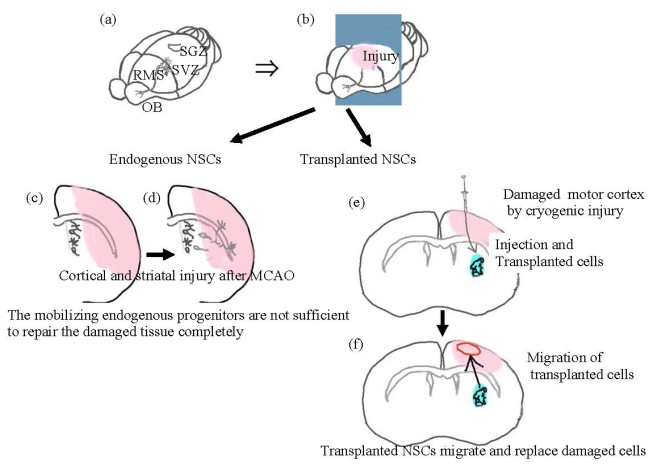
Schematic representation of neural cell migration in mouse brain. (**a** and **b**) Stereographic drawing of the brain and three dimensional positions of the subventricular zone (SVZ), subgranular zone (SGZ), rostral migratory stream (RMS) and olfactory bulb (OB). Continuous generation of neural cells is shown in both SVZ of the lateral ventricle and SGZ of the dentate gyrus in the mouse hippocampus; (**c** and **d**) Endogenous neural stem cell (NSC) migration to facilitate regeneration of the damaged area in the middle cerebral artery occlusion (MCAO) model. Migratory pathway of the endogenous neural cells generated in SVZ is shown. They migrate to the injured area directly or through RMS and OB. However, numbers of the endogenous cells are insufficient to enable the repair of the damaged tissue; (**e** and **f**) Neural cell migration after ES cell derived NSC transplantation in the hemiplegic mouse with brain injury. We injected ES cell derived neural stem/progenitor cells into the periventricular region of the striatum. The transplanted cells migrated to the injured motor cortex and located diffusely over the cortex. We speculated that the grafted cells replaced and regenerated the damaged cortex, leading to recovery of the hemiplegia.

Cerebral vascular diseases often cause severe neurological dysfunctions with high disability and mortality [1]. There is accumulating evidence that supports effectiveness of neural tissue transplantation on the recovery of neurological dysfunctions in experimental stroke models [1–6]. In rodents, endogenous neural stem cells are enriched in the subventricular zone (SVZ) [7] and the subgranular zone (SGZ) [8] even in adult brains. Neural stem cells in the SVZ migrate to the olfactory bulb (OB) through the rostral migratory stream (RMS) and they differentiate into neural cells [9]. This migration of stem cells is called chain migration because of its histological features. Neural stem/progenitor cells have high abilities to proliferate and to differentiate into several neural cell types in the CNS [10–12]. Therefore, endogenous neural stem/progenitor cells may be responsible for the spontaneous functional recovery frequently observed in the damaged CNS of mice. In brain injury models of mice, astrocytes around the injured area activate neural stem cells in SVZ and SGZ, and promote them to migrate to the injured area, where they differentiate into mature neural cells (Figure 1) [6,13,14]. However, the number of endogenous neural stem/progenitor cells is not sufficient to repair the damaged brain efficiently.

The neural cells derived from the endogenous neural stem cells migrating from SVZ in the injured area occupy only 0.2% of the dead neurons in the middle cerebral artery occlusion (MCAO) model [13]. The human brain has RMS that consists of similar cells to the rodent brain including OB [15]. The activity of cells in RMS is relatively lower in humans than in rodents. It suggests that the cell replacement rate in human OB is much lower than in rodents. Moreover the weight of human OB is below 0.1% of the whole human brain, whereas rodent OB occupies more than 20% of the total rodent brain [16]. These findings suggest that the regenerative and proliferating potential of the cells in OB are much lower in humans than in rodents [15].

Transplantation of exogenous neural cells derived from ES and induced pluripotent stem (iPS) cells has the possibility to provide sufficient numbers of neural cells to the damaged tissue, leading to the restoration of lost motor functions. In our hemiplegic model of mice, transplantation with both mouse and monkey ES cell derived neural stem/progenitor cells improved motor functions [4,5,17]. The neural graft derived from the ES cells migrated to the injured area and expressed neural cell adhesion molecules which mediated homophilic binding [5]. We speculate that both cell-to-cell and cell-to-soluble factor interactions are essential for the grafted neural stem/progenitor cells to differentiate toward neural cells suitable for their neighboring environment and to form new neural circuits with the damaged host neurons.

Chemokines are small polypeptides consisting of about 100 amino acids, initially identified as molecules which belong to a subtype of cytokines produced from immune cells, and contribute to the maturation and trafficking of leukocytes. Chemokines and their receptors play an important role in neural cell migration and they are constitutively expressed in glial and neural cells in the CNS.

Chemokines are categorized into four groups (CXC, CC, C and CX3C chemokines) by the number and position of the conserved cysteine residues in their amino termini. Chemokine receptors are categorized into four groups (CXCRn, CCRn, XCRn and CX3CRn), each corresponding to the respective chemokine nomenclature above, and belong to seven-transmembrane-domain G-protein coupled receptors. Several chemokines are involved in neural formation in both ontogenic development and tissue regeneration through their interaction with neural cell surface molecules [18–20].

In our hemiplegic model, stromal cell derived factor 1 (SDF1) and some neural cell associated adhesion molecules, such as L1 cell adhesion molecule (L1CAM), neural cell adhesion molecule (NCAM) and N-cadherin, were associated with migration and maturation of the neural stem/progenitor cells derived from monkey ES cells. Here, we review chemokine regulation of neural regeneration after ES cell derived neural stem/progenitor cell transplantation.

## 2. Chemokine Expressions in the CNS

In the human physiological condition, several chemokines such as monocyte chemoattractant protein 1 (MCP1), cutaneous T cell-attracting chemokine (CTACK), SDF1 and fractalkine are expressed on neural cells and astrocytes of the brain and on primary cells isolated from the CNS (Table 1). The expressions of interferon-γ-inducible protein of 10 kDa (IP10), SDF1 and fractalkine are enhanced in several human diseases such as HIV infection and multiple sclerosis. Certain chemokines play a key role in brain development (Table 2). Regulated upon activation, normal T cell expressed and secreted (RANTES) and SDF1 evoke neural cell migration, while Growth-regulated oncogene α (GROα), MCP1, Macrophage inflammatory protein 1α (MIP1α), RANTES and SDF1 affect astrocyte proliferation.

**Figure 2:**
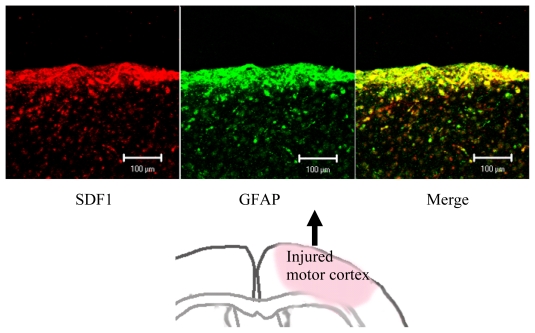
Stromal cell derived factor 1 (SDF1) expression by glial fibrillary acidic protein (GFAP) positive cells in the damaged motor cortex of mice. Motor cortex was injured and stained with anti-SDF1 antibody (Red) and anti-mouse GFAP antibody (Green) in our hemiplegic model. Almost all GFAP positive cells expressed SDF1.

Upregulations of GROα [38], MCP1 [39], MIP1α [40] and SDF1 [41,42] are shown in the hemiplegic models (Figure 2) [5,41,42], and in patients with cerebral vascular diseases. These chemokines are suggested to have neuroprotective functions [42–44]. Thus, the same chemokine induces both CNS inflammation and regeneration. Judging from the poor clinical outcomes with stroke patients, the regenerative ability of the chemokine is much less than the inflammation-inducing destructive ability in humans.

## 3. Transplanted Neural Cells and Chemokines

**Figure 3:**
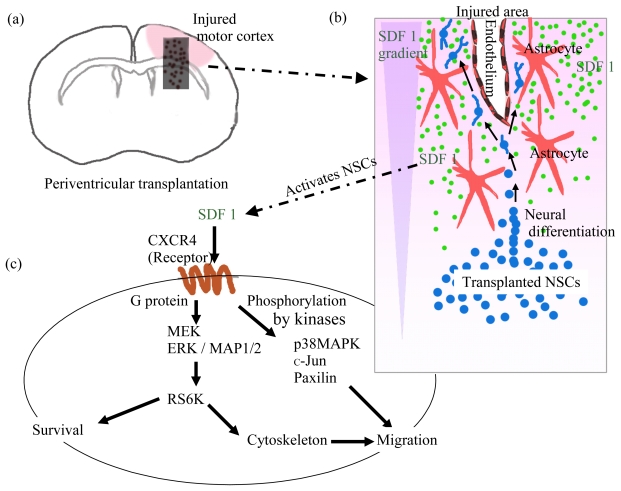

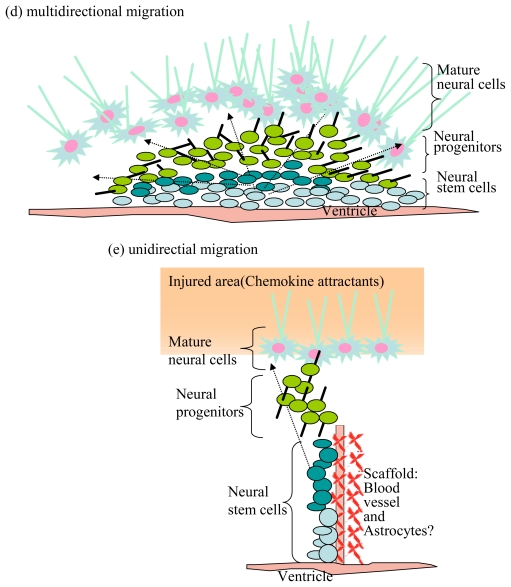
Involvement of chemokines/chemokine receptors in the neural regeneration in an experimental hemiplegic model with neural cell transplantation. (**a**,**b**) Astrocytes and vascular endothelial cells in the injured area (pink colored) produce several chemokines (e.g., SDF1). After expressing chemokine receptors (e.g., CXCR4), the neural stem/progenitor cells react with the chemokines and then start to move along with the concentration gradient of the chemokines, whose concentration is the highest at the injured area; (**c**) The SDF1/CXCR4 interaction on the neural cell graft activates several signaling molecules including p38MAPK, ribosomal S6 kinase, c-Jun and paxillin. The interactions are followed by various cell activities, such as proliferation, chemotaxis and migration with conformational changes of cytoskeleton for neurite outgrowth; (**d**) Conceptional classification of neural cell migration in mice with transplantation. One is multidirectional migration and the other is unidirectional migration of neural stem/progenitor cells. Multidirectional migration means that neural stem/progenitor cells accumulated in a region migrate in all directions; (**e**) Unidirectional migration means that neural stem/progenitor cells continuously move on in one direction, dependent on the lower to higher concentration gradient of the chemokines. Cells in the unidirectional migration look like a chain of cells in histological examination.

After brain injury, astrocytes respond to some signals from the injured area and produce various molecules which provoke neural stem/progenitor cell proliferation, migration and differentiation to start regeneration of the damaged tissue (Figure 3a,b) [42–44]. Astrocytes produce several inflammatory chemokines, such as SDF1, MCP1, MIP1α, MIP1β, RANTES and fractalkine [18,20]. Neural stem/progenitor cells express chemokine receptors, such as CCR2, CCR5, CXCR3, and CXCR4 [45]. It has been reported that CCR2 (the receptor of MCP1) and CXCR4 (the receptor of SDF1) are involved in the migration of endogenous neural stem cells to the injured area in mice [44,46]. Interaction between SDF1 and CXCR4 activates several signaling molecules in neural stem cells including p38MAPK, ribosomal S6 kinase, c-Jun and paxillin. The interactions are followed by various cell activities, such as proliferation, chemotaxis and migration with conformational changes of cytoskeleton for neurite outgrowth. The SDF1/CXCR4 interaction on the neural cell graft activates several signaling pathways including mitogen-activated protein kinase (MAPK). Various cellular responses including proliferation and migration of the neural cell graft are induced by the MAPK activation. As for migration, activated p38MAPK is involved in actin reorganization essential for cell migration. The ribosomal S6 kinase (RS6K), which is activated by extracellular signal-regulated kinases (ERK), provokes the phosphorylation of cytoskeletal molecules which are mandatory for cell migration and neurite elongation. MAP/MEK kinase 1 (MEKK1) is essential for cell migration, because the c-Jun *N*-terminal kinase (JNK), which is phosphorylated by MEKK1, phosphorylates the focal adhesion adaptor molecule, paxillin, important for cell migration. Collectively, accumulation of the phosphorylation and subsequent activation events following SDF1/CXCR4 interaction brings about neural cell migration, observed in mice with neural cell grafts (Figure 3c) [47–50]. SDF1 stimulates cortical astrocyte proliferation through Src-ERK1/2 activation [36].

SDF1 and CXCR4 are highly expressed in the embryonic brain. Mice with congenital deficit of CXCR4 or SDF1 shared the same pathological development, hypoplasia of hippocampal external granule cell layer [51,52]. Recent analysis using CXCR4 transfected neural progenitor cells revealed that SDF1/CXCR4 regulated adult neural progenitor cell motility but not differentiation [53]. SDF1 utilizes both CXCR4 and CXCR7 for its receptor. SDF1 activates Galpha (i1) protein-dependent signaling pathway through CXCR4. Through CXCR7, SDF1 does not activate Galpha (i1) signaling pathway but activates the MAP kinase pathway [54]. In mouse development, CXCR7 also contributes to cortical interneuron migration. Mice deficient either in CXCR4 or CXCR7 show similar phenotypes. However, there is a distinct difference in the interneuron tangential and radial migration motility between CXCR4 and CXCR7 deficient mice during the early embryonic stage [55]. We think that further studies are needed to clarify the relation between these molecules in the regeneration of damaged CNS after transplantation.

When we conducted monkey ES cell derived neural cell transplantation into hemiplegic mice with brain injury (Figure 1), a significant recovery of motor functions was observed [3]. We found unidirectional migration of the neural cells from the grafted periventricular region toward the damaged motor cortex where SDF1 was expressed extensively. This migration resembled so-called chain migration, physiologically shown in embryonic brain development and adult forebrain (Figure 3b,e).

Using a microchemotaxis assay *in vitro*, we showed that the migration ability of mouse and monkey ES cell derived neural stem/progenitor cells depended on the concentration gradient of SDF1. It has been shown that by the blocking of CXCR4 signaling by AMD3100 *in vitro* and *in vivo*, an antagonist of CXCR4 [5,56], inhibited migration of the neural stem/progenitor cells. Migration of the neural stem/progenitor cells was not affected by other chemokines, such as MCP1, CTACK, RANTES, fractalkine and MIP1α. The ES cell derived neural stem/progenitor cells expressed CXCR4 but did not express CCR2 or any other major chemokine receptors [5].

In another *in vitro* study of our own, we found that ES cells differentiated efficiently into neural cells in the presence of SDF1, whereas MCP1 and other major chemokines did not affect their differentiation. We think that migration and subsequent maturation of the neural stem/progenitor cells transplanted to the damaged brain are mainly caused by SDF1 secreted from glial cells accumulating around the injured area (Figures 2 and 3b) [5].

We found that ES cell derived neural stem/progenitor cells migrated as a cell aggregate from the periventricular region of the striatum where they had been injected in the damaged motor cortex. The migration was inhibited by the administration of AMD3100, suggesting that the cells were guided by the concentration gradient of SDF1 which was secreted by glial cells accumulated in the damaged cortex. Migration of the grafted neural stem/progenitor cells resembled so-called chain migration, but not radial (multidirectional) migration in the recipient brain (Figure 3d) [5].

CCR2 deficient mice do not show any abnormality of neural stem/progenitor cells throughout the embryonic CNS development. Low expressions of MCP1 and CCR2 molecules on the CNS are physiologically recognized in the mice throughout their lives. Redundancy of chemokines/cytokines may explain the lack of abnormality [20].

## 4. Neural Stem/Progenitor Cells and Neural Cell Associated Adhesion Molecules

Eventually, the grafted cells migrate into the superficial layer of the injured motor cortex and re-connect the pyramidal tract which has once been damaged with their extended axons. Migration of the endogenous neural cells in the adult forebrain, including RMS, needs interactions with neighboring cells via neural cell associated adhesion molecules and other cell surface molecules.

The polysialylated form of the neural cell adhesion molecule (PSA-NCAM) is one of the homophilic binding cell adhesion molecules. Homophilic PSA-NCAM interaction between endogenous neural stem/progenitor cells emerging from SVZ and surrounding neural cells, which have been existing there, is important to form the chain migration (or tangentinal migration) of RMS [57,58]. The neural cells in chain migration are surrounded by a microenvironment mainly consisting of astrocytes, with which the neural stem/progenitor cells interact to promote their migration (Figure 3b) [59].

Neural cell associated adhesion molecules, such as L1CAM and NCAM, and N-cadherin are important for axon elongation [60,61]. L1CAM and NCAM are members of the immunoglobulin superfamily and they are widely expressed in neural tissues during development. Both L1CAM and NCAM mediate homophilic and heterophilic adhesion [62]. Cell adhesion molecules are assigned an important role in the cytoskeletal and transcriptional event during neurite outgrowth [60]. L1CAM plays a role in neurite extension and NCAM is important for the cone protrusive growth of axon [63]. L1CAM deficient mice have enlarged ventricles and severe hypoplasia of the corticospinal tract [64]. NCAM deficient mice show a primary defect in embryonic neural cell migration and subsequent defects in axon growth and fasciculation [65]. Mice deficient in N-cadherin show that neuroepithelial and radial glial cells do not expand their processes to span the distance and therefore terminate them in the middle zone of the cortex, suggesting that N-cadherin contributes to neurite outgrowth and synaptic connection [66].

In our study, ES cell derived neural stem/progenitor cells started to express NCAM and L1CAM mRNAs 4 h after SDF1 stimulation *in vitro* [5]. The cells grafted to mouse brain started to express NCAM, L1CAM and N-cadherin simultaneously soon after transplantation, and formed homophilic and heterophilic intercellular bindings and *de novo* neural network at the damaged cortex 28 days after transplantation [5]. It was possible that L1CAM, NCAM, and N-cadherin induced by SDF1 contributed to regenerating neural network by promoting the extension of axons/neurites in the damaged cortex [67,68].

## 5. Conclusions

SDF1 and several neural cell adhesion molecules play a role in migration and differentiation of the grafted neural stem/progenitor cells and subsequent neural network reconstruction in the damaged brain. We found that SDF1 was one of the most important molecules among other chemokines tested so far for the regulation of the neural stem/progenitor cell migration and the formation of neural network. Endogenous glial cells around the injured area mainly secreted SDF1. NCAM was expressed on the transplanted neural cell after reaching the damaged cortex. SDF1 induced to express neural cell associated adhesion molecules, which in turn helped promote appropriate differentiation of neural stem/progenitor cells and subsequent regeneration of neural network *in vivo*.

Our hemiplegic mouse model served as a basis to understand the molecular mechanisms governing neural regeneration after transplantation, and indicated the importance of SDF1 and the neural adhesion molecules for the recovery of motor functions.

## Supplementary Material



## Figures and Tables

**Table 1:** Expression of chemokine molecules in human brain cells.

Chemokine	Receptor	Distribution
**Physiological**		
*In vivo*		
MCP1/CCL2	CCR2	Fetal and adult brain: Neurons [21]
CTACK/CCL27	CCR10	Adult brain: Neurons [22]
SDF1/CXCL12	CXCR4	Adult brain: Neurons [23]
Fractalkine/CX3CL1	CX3CR1	Adult brain: Astrocytes [24]
*In vitro*		
MCP1/CCL2	CCR2	Primary astrocyte culture [25]
MIP1α/CCL3	CCR3	Primary astrocyte culture [25]
MIP1β/CCL4	CCR5	Primary astrocyte culture [25]
RANTES/CCL5	CCR5	Primary astrocyte culture [25]
SDF1/CXCL12	CXCR4	Primary fetal neural cell culture [23]
Fractalkine/CX3CL1	CX3CR1	Primary neural and glial cell culture [26]
**Pathological**		
MCP1/CCL2	CCR2	Astrocytes in HIV encephalitis [27]
RANTES/CCL5	CCR5	Astrocytes in HIV encephalitis [27]
IP10/CXCL10	CXCR3	Neurons in HIV encephalitis [28]
SDF1/CXCL12	CXCR4	Neurons and Astrocytes in AIDS dementia [23]
Fractalkine/CX3CL1	CX3CR1	Astrocytes in multiple sclerosis [24] Neurons in HIV encephalitis [29]

MCP1: monocyte chemoattractant protein 1; CTACK: cutaneous T cell-attracting chemokine; SDF1: stromal cell derived factor 1; MIP1: Macrophage inflammatory protein 1; RANTES: regulated upon activation, normal T cell expressed and secreted; IP10: interferon-γ-inducible protein of 10 kDa.

**Table 2:** Chemokine expression in brain development.

Chemokine	Receptor	Target Cells
**Cell Migration**		

RANTES/CCL5	CCR5	Neurons [30]
SDF1/CXCL12	CXCR4	Cerebellar granule neurons [31] Cortical neural progenitors [32] Dentate gyrus granular neurons [33]
MCP1/CCL2	CCR2	Astrocytes [34]
**Cell Proliferation**		

MIP1α/CCL3	CCR3	Astrocytes [34]
RANTES/CCL5	CCR5	Astrocytes [25]
GROα/CXCL1	CXCR2	Astrocytes, Oligodendrocyte precursors [35]
SDF1/CXCL12	CXCR4	Astrocytes [36,37]
