# Supplementary material for: Role of SDF1/CXCR4 Interaction in Experimental Hemiplegic Models with Neural Cell Transplantation
Source: Int J Mol Sci. 2012 Feb 28;13(3):2636–49. doi: 10.3390/ijms13032636 (PMC3317678; doi:10.3390/ijms13032636)

Table 1 Expression of chemokine molecules in human brain cells

Chemokine	Receptor	Distribution
**Physiological**		
In vivo		
MCP-1/CCL2	CCR2	Fetal and adult brain: neurons[56]
SDF-1/CXCL12	CXCR4	Adult brain: neurons[57]
Fractalkine/CX3CL1	CX3CR1	Adult brain: astrocytes[58]
In vitro		
MCP-1/CCL2	CCR2	Primary astrocyte culture[59]

MIP-1α/CCL3	CCR3	Primary astrocyte culture[59]
MIP-1β/CCL4	CCR5	Primary astrocyte culture[59]
RANTES/CCL5	CCR5	Primary astrocyte culture[59]
IL-8/CXCL8	CXCR2	Primary astrocyte culture[59]
SDF-1/CXCL12	CXCR4	Primary fetal neural cell culture[57]
Fractalkine/CX3CL1	CX3CR1	Primary neural and glial cell culture[60]
**Pathological**		
IP-10/CXCL10	CXCR3	Neurons in HIV encephalitis[61]
SDF-1/CXCL12	CXCR4	Neurons and astrocytes in AIDS dementia[57]
Fractalkine/CX3CL1	CX3CR1	Astrocytes in multiple sclerosis[58]
		Neurons in HIV encephalitis[62]

Table 2: Chemokine expression in brain development

Chemokine	Receptor	Target cells
**Cell migration**		
RANTES/CCL5	CCR5	neurons[63]
SDF-1/CXCL12	CXCR4	cerebellar granule neurons[64]
		cortical neural progenitor[65]
		dentate gyrus granular neurons[66]
**Cell proliferation**		
Gro-α/CXCL1	CXCR2	astrocytes, oligodendrocyte precursor[67]

MCP-1/CCL2	CCR2	astrocytes[68]
MIP-1α/CCL3	CCR3	astrocytes[68]
RANTES/CCL5	CCR5	astrocytes[59]
SDF-1/CXCL12	CXCR4	astrocytes[33,69]

Figure 1. Schematic representation of neural cell migration in mice

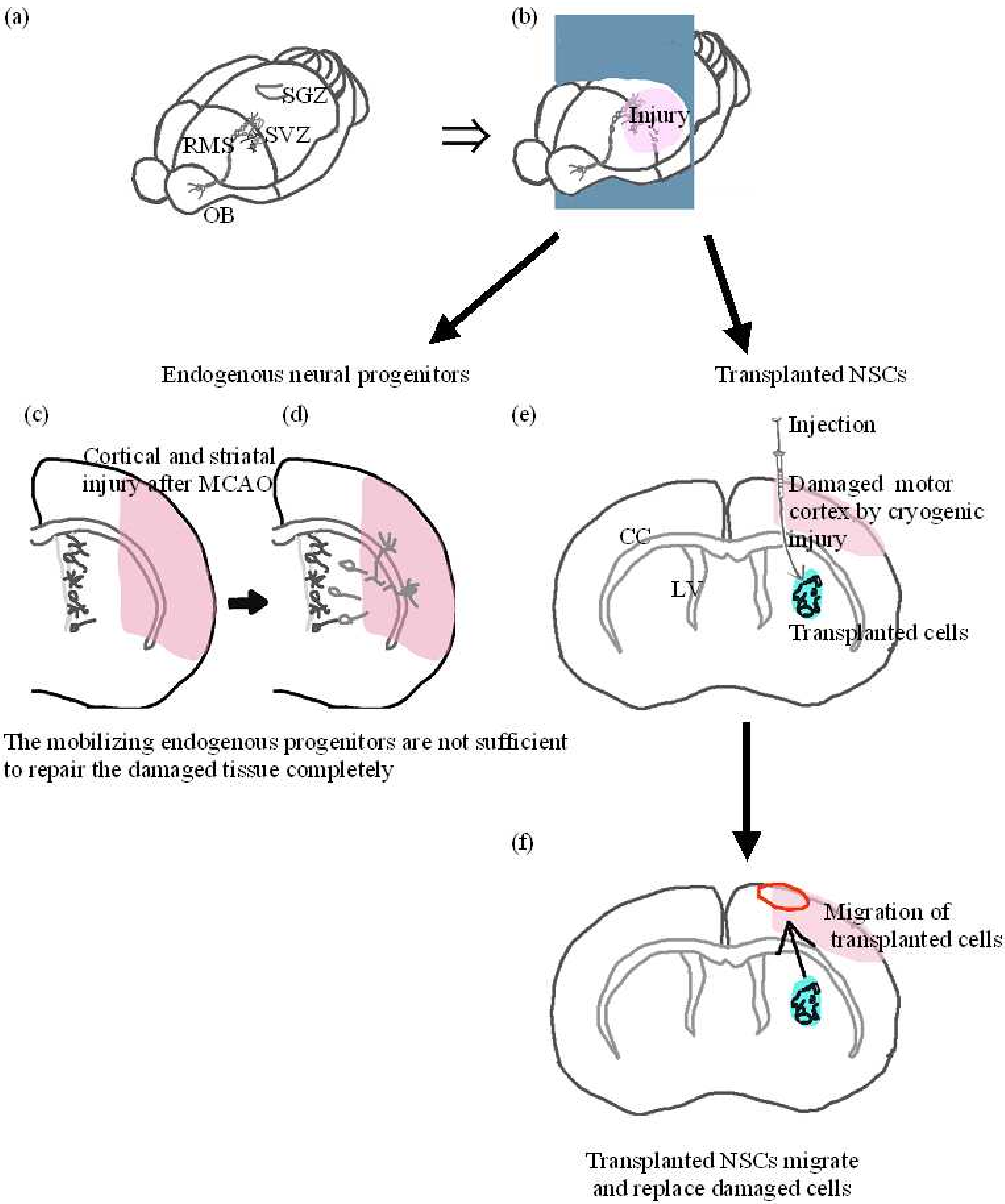

Figure 2. SDF1 expression by glial fibrillary acidic protein (GFAP) positive cells in the damaged motor cortex in mice

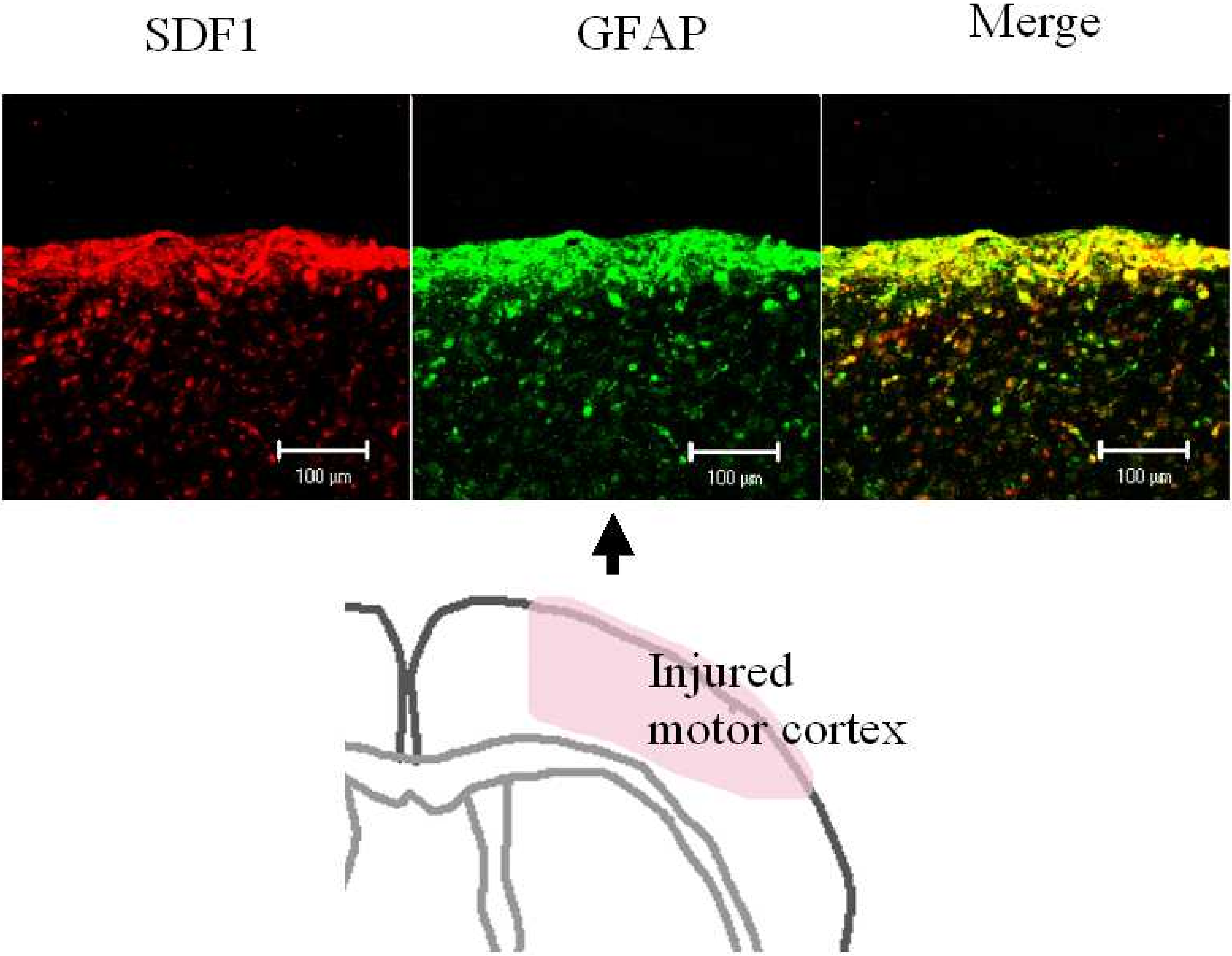

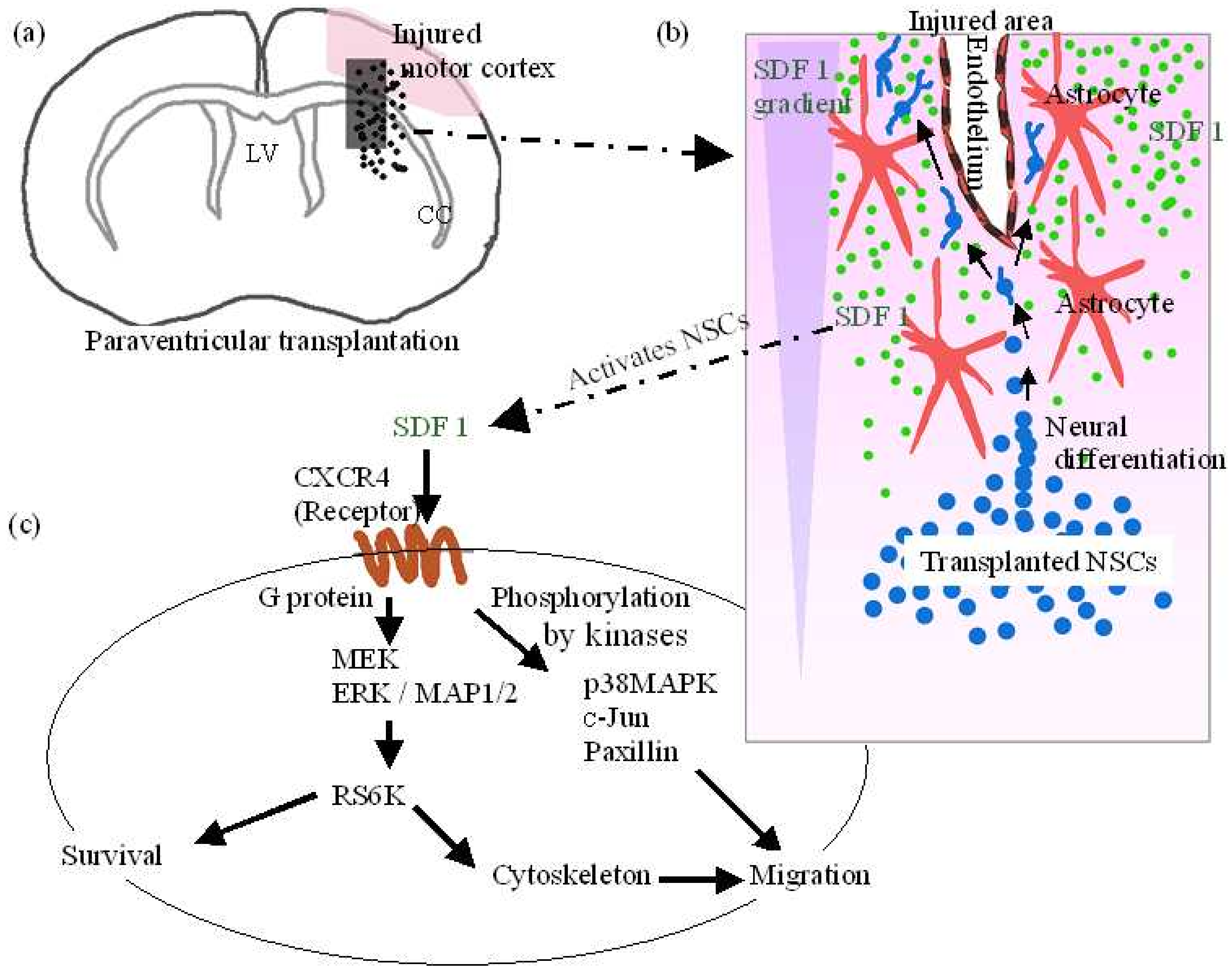

Figure 3. Involvement of chemokines/chemokine receptors in the neural cell migration after cell transplantation in injured mouse motor cortex

Figure 3. Involvement of chemokines/chemokine receptors in the neural cell migration after cell transplantation in injured mouse motor cortex

(d) Radial migration

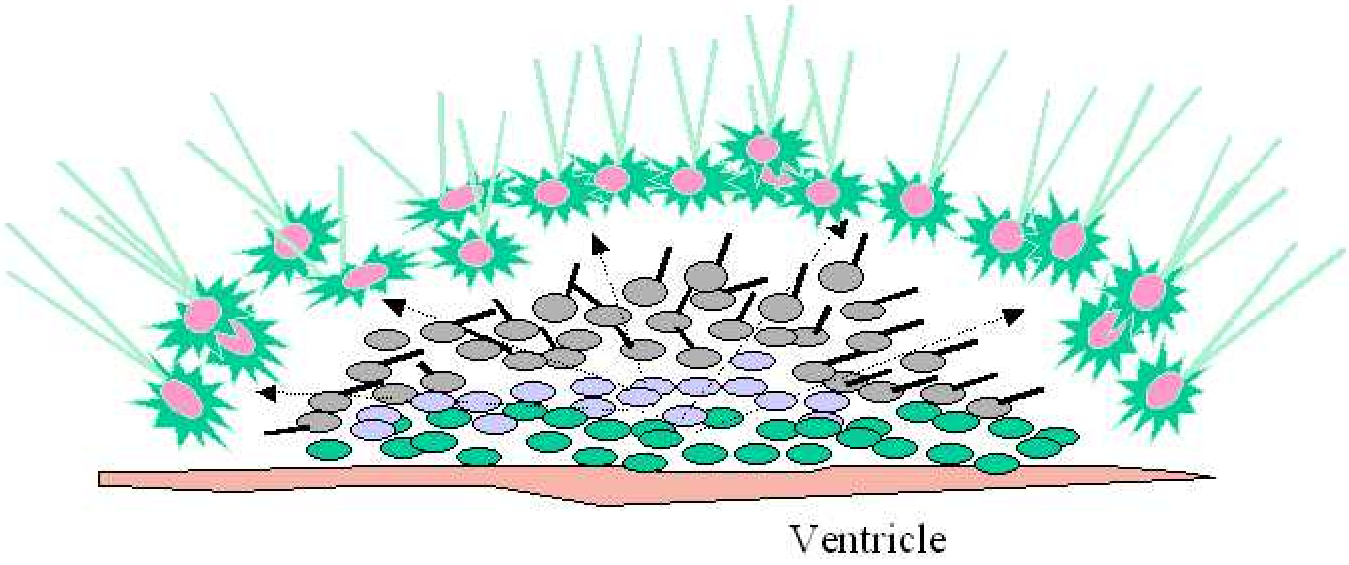

(e) Tangential migration

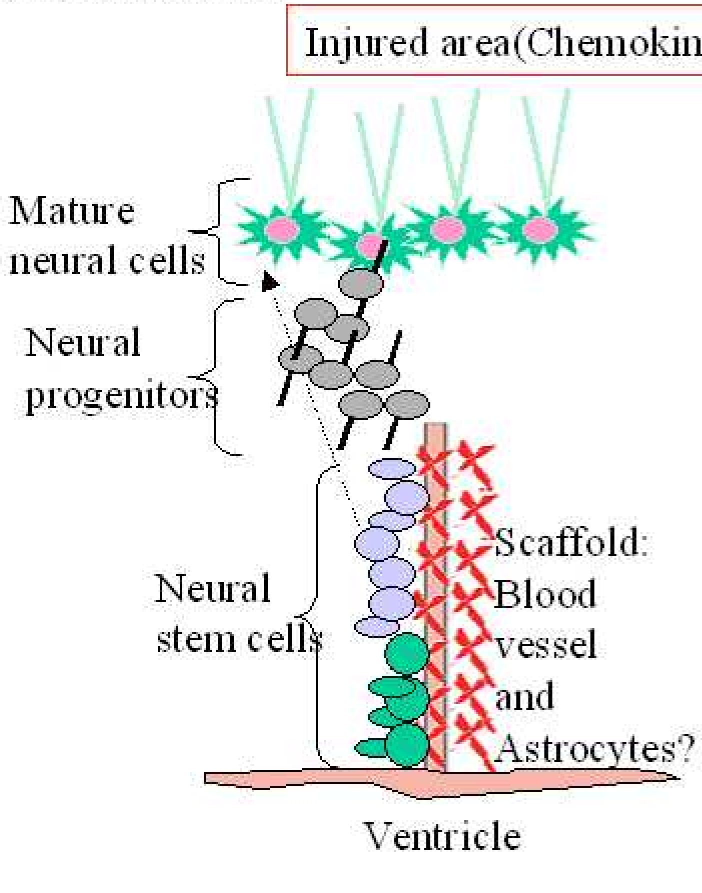

Supplement: Supplementary file 1 [file ijms-13-02636-s001.pdf]
